# Understanding uptake of an intervention to accelerate antiretroviral therapy initiation in Uganda via qualitative inquiry

**DOI:** 10.1002/jia2.25033

**Published:** 2017-12-05

**Authors:** Fred C Semitala, Carol S Camlin, Jeanna Wallenta, Leatitia Kampiire, Richard Katuramu, Gideon Amanyire, Jennifer Namusobya, Wei Chang, James G Kahn, Edwin D Charlebois, Diane V Havlir, Moses R Kamya, Elvin H Geng

**Affiliations:** ^1^ Department of Medicine Makerere University College of Health Sciences Kampala Uganda; ^2^ Makerere University Joint AIDS Program (MJAP) Kampala Uganda; ^3^ Infectious Diseases Research Collaboration Kampala Uganda; ^4^ Division of HIV, ID and Global Medicine Department of Obstetrics, Gynecology & Reproductive Sciences Department of Medicine University of California San Francisco CA USA; ^5^ Philip R. Lee Institute for Health Policy Studies Department of Epidemiology and Biostatistics, and Global Health Sciences University of California San Francisco CA USA; ^6^ Division of HIV, ID and Global Medicine Department of Medicine University of California San Francisco CA USA

**Keywords:** Implementation Research, Stepped Wedge, PRECEDE framework, PLHIV peer counsellors, ART initiation, Theoretical Domain Framework

## Abstract

**Introduction:**

The Streamlined Antiretroviral Therapy Initiation Strategy (START‐ART) study found that a theory‐based intervention using opinion leaders to inform and coach health care providers about the risks of treatment delay, provision of point of care (POC) CD4 testing machines (PIMA) and reputational incentives, led to rapid rise in ART initiation. We used qualitative research methods to explore mechanisms of provider behaviour change.

**Methods:**

We conducted in‐depth interviews (IDIs) with 24 health care providers and nine study staff to understand perceptions, attitudes and the context of changes in ART initiation practices. Analyses were informed by the Theoretical Domains Framework.

**Results:**

Rapid dissemination of new practices was enabled in the *environmental context* of an existing relationship based on communication, implementation and accountability between Makerere University Joint AIDS Program (MJAP), a Ugandan University‐affiliated organization that provided technical oversight for HIV service delivery at the health facilities where the intervention was implemented, and a network of health facilities operated by the Uganda Ministry of Health. Coaching carried out by field coordinators from MJAP strengthened influence and informal accountability for carrying out the intervention. Frontline health workers held a pre‐existing strong sense of *professional identity*. They were proud of attainment of new knowledge and skills and gratified by providing what they perceived to be higher quality care. *Peer counsellors*, who were not explicitly targeted in the intervention design, effectively substituted some functions of health care providers; as role models for successful ART uptake, they played a crucial role in *creating demand* for rapid ART initiation through interactions with patients. Point of care (POC) CD4 testing enabled immediate action and relieved providers from frustrations of lost or delayed laboratory results, and led to higher patient satisfaction (due to reduced costs because of ability to initiate ART right away, requiring fewer return trips to clinic).

**Conclusions:**

Qualitative data revealed that a multicomponent intervention to change provider behaviour succeeded in the context of strong institutional and individual relationships between a University‐affiliated organization, government facilities, and peer health workers (who acted as a crucial link between stakeholders) and the community. Fostering stable institutional relationships between institutional actors (non‐governmental organization (NGOs) and ministry‐operated facilities) as well as between facilities and the community (through peer health workers) can enhance uptake of innovations targeting the HIV cascade in similar clinical settings.

## 1 Introduction

Understanding whether effects observed in one setting will have the same effects in another setting, often referred to as generalizability, is a critical challenge for implementation science's goal to identify widely useable strategies to address HIV 1. For example, using multiple counselling sessions to prepare patients for ART 2, 3, 4, 5 appear to be beneficial in some studies 3 but not others 6. These differences may depend on contextual factors; for example, the level of social support in the community, extent of HIV‐related stigma or quality of care services. Yet, there is a tremendous need for interventions that work at scale and across such diverse settings. The Streamlined antiretroviral therapy (START‐ART) (NCT01810289) study tested a strategy using opinion leaders, point of care CD4 and a reputational incentive to accelerate initiation of treatment. The intervention yielded large effects: 79.6% initiated ART by two weeks after eligibility in the intervention compared to 37.7% in the control 7. Understanding how this intervention worked and whether this intervention would have similar effects in different settings can help advance an evidence‐based global response to HIV.

Little consensus exists, however, for evaluating the generalizability of interventions such as the START‐ART. Many interventions are at target facilities 8, and therefore repeated evaluation can be an expensive undertaking. Understanding how an intervention works, both within particular contexts and through particular mechanisms, offers an alternative way to obtain information that can enable informed assessments of generalizability. Interventions that have shown increases in ART access and retention to date, however, have not always conceptualized or reported the context and/or the mechanism of effect. For example, community adherence groups have received much attention 9. If these groups work only via reducing transport and opportunity costs, then dispensing six months of pills at each refill would have the same effect. If these groups act through social support, facilities with buffer stock would not adequately address the gaps. Likewise, SMS messages for adherence may act as simple reminders or alternatively convey encouragement and engagement 10, 11.

Qualitative research methods are particularly useful for undertaking in‐depth inquiry into potential mechanisms through which various interventions may improve HIV care cascade outcomes. How interventions work in implementation settings is often composed of both anticipated as well as unanticipated mechanisms. In this paper, we used qualitative methods to understand the context and mechanism of the effect, with the ultimate goal of understanding in greater depth the potential for generalizability of the intervention.

## 2 Methods

### 2.1 Study context

**Figure 1:**
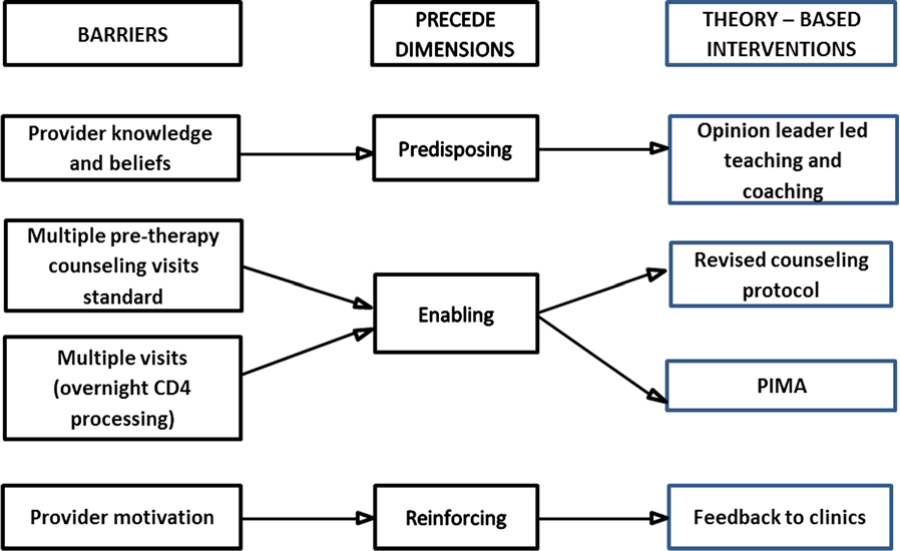
An illustration of how the START ART study used the PRECEDE framework concepts of predisposing, enabling and reinforcing factors to enhance ART initiation in Uganda.

START‐ART, a stepped‐wedge cluster randomized trial, was conducted between May 2013 and August 2015. The START‐ART intervention consisted of three components informed by PRECEDE framework concepts of predisposing, enabling and reinforcing factors 12 to address barriers to ART initiation (Figure 1). First, we used opinion leader‐led interactive training sessions with HIV care providers, which conveyed recent scientific evidence regarding effects of rapid ART initiation on patient survival, and introduced a revised counselling approach. This approach relaxed strict requirements for treatment supporters, and emphasized that patients should be assessed for ART readiness at each visit including first clinic registration. Second, we introduced a point‐of‐care (POC) PIMA^™^ CD4 test machine to each clinic to enable health care workers to offer “real time” results. Finally, we provided biannual feedback to the facilities that involved presentation of the clinic ART initiation rates as compared with other clinics to motivate poorly responsive sites. The study was implemented through the Makerere University Joint AIDS Program (MJAP), a Ugandan organization owned by Makerere University and supported by The U.S. President's Emergency Plan for AIDS Relief (PEPFAR). MJAP pays salaries of programme managers and technical staff, but many health facility managers and frontline health care providers’ salaries are paid by the government of Uganda. A central management team at MJAP oversees HIV care and treatment policies, and conducts planning and resource‐sharing meetings involving the technical team and the district leadership, in which decisions are made and then communicated to facility managers. This multisectoral approach was established in order to strengthen the provision of HIV services, in a setting that has been constrained by shortages of trained human resources, inadequate equipment and barriers to dissemination of up to date knowledge on HIV care. The health facilities supported by MJAP range from rural health centres to subdistrict level facilities as well as three University teaching hospital‐based clinics, all of which provide the full continuum of HIV services from HIV testing to long‐term retention in HIV care.

### 2.2 Qualitative study design

The qualitative study was conducted in the five health care facilities that comprised wave three of START‐ART. Data were collected two months prior to intervention rollout, and 7–12 months after the intervention was introduced in the clinics between 31 March and 10 April 2014. These clinics were representative of large and small clinics across the geographic area for the trial; two were urban, within the national referral and teaching hospital (Mulago), while the rest were in rural settings. The analyses for this paper used data collected from providers, peer counsellors who are people living with HIV (PLHIV), study staff and programme implementers.

### 2.3 Sampling

The sample of health care providers was purposively selected to be composed of all of the individuals who were engaged in or overseeing ART and HIV care delivery and counselling in the facilities. These included two nurses, five counsellors and six medical officers; as well as nine PLHIV engaged in care (peer counsellors), distributed across five health facilities (the numbers of these cadres varied from one clinic to another). A total of 22 pre‐intervention and 19 post‐intervention provider interviews were conducted. The providers included in the pre‐intervention were also interviewed in the post‐intervention, with the exception of three staff who had left facilities where the pre‐intervention interview was conducted. Key informant interviews were conducted with two study coordinators, two study counsellors and one clinic manager involved in START‐ART implementation, and with the Executive Director and two senior technical staff at MJAP.

### 2.4 Data collection

Data were derived from in‐depth semi‐structured interviews (IDIs), using guides for health care provider interviews and key informant interviews that were designed to assess evidence for changes in providers’ attitudes and practices, and factors in the health systems context, that could facilitate uptake of accelerated ART delivery. For provider post‐intervention interviews, summary reports of pre‐intervention transcripts were prepared in order to tailor follow‐up questions. The guide included questions about providers’ perceptions of the intervention, and how it might have affected providers’ working conditions for the provision of ART. Interviews were conducted by two research assistants and a study coordinator who were trained by the lead investigator for the qualitative study. Key informant interviews were conducted by the lead investigator. Research team members were native speakers of local languages and conducted interviews in participants’ preferred language. The interviews were recorded and recordings were transcribed and translated into English, and loaded into ATLAS.ti software (version 7.1.7; GmbH, Berlin, Germany) for coding and analysis.

### 2.5 Data analysis

Our analysis approach drew upon constructivist grounded theoretical methods 13, and also was informed by the Theoretical Domains Framework (TDF) 14 an implementation science framework for understanding the uptake of new evidence‐based practices, which posits four dimensions of influence on this process, which operate at individual, team, and organizational levels: *environmental context and resources*,* social influences*,* social/professional role and identity* and *behavioural regulation* (this dimension is inclusive of the influence of interventions to manage energy, emotions, attention and behaviour, e.g. implementation intentions) 15. (Table 1). An initial list of a priori codes was developed by the lead investigator for the qualitative study and the research coordinator based on the major thematic categories in theory‐informed interview guides. Codes were applied by the research coordinator and members of the data collection team, who were trained in focused coding methods by the lead investigator. The coding framework was discussed and iteratively refined during the data collection process, following rounds of review of interview transcripts. Codes were then extracted as query reports (queries are search expressions, and query reports are excerpts of transcripts falling within specific codes used in search terms), which were used to review and reduce data into coherent analytical categories. An analysis matrix was developed by the lead investigator and research coordinator to summarize themes emergent in the data, along with illustrative rich quotes categorized by data source.

**Table 1:** Uptake of an intervention to accelerate ART initiation in Uganda: illustrative quotes, by theorized categories of influence in the TDF

TDF Dimension	Emergent Themes
Environmental context and social influences	Good lines of policy communication from Ministry of Health to frontline providers through MJAP: “… for the ART clinic, everybody has a heart for what the clinic is. Working with MJAP, they will take on any extra responsibility that comes, along with any policy changes. Ministry of Health regularly changes policies; they [MJAP] incorporate, even data tools. So, I feel they are positive.” *Medical officer at a rural high volume HIV clinic* Clear oversight and a sense of accountability to and recognition by MJAP and Ministry of Health leadership: “The ART clinic is a clinic that has grown to a level of local community, [at] the district but even at national level… [is] being appreciated, being recognized for what it is doing. So am sure if really the basic equipment, adequate staffing is offered, adequate technical support, this is a model of accepted standards of service provision.” (Medical officer at a rural high volume HIV clinic) Strong network connections between MJAP intervention staff and frontline providers, facilitating a sense of solidarity and trust: “…they [the health care providers at study clinics] were looking at us [research team members] as more like colleagues who have come to help in the system… I arrive at 2:00, and there are maybe about five patients. I sit there and do a bit [of work] and say, “how was the clinic? Is it okay? Let me help you with these five patients.” So with that one, it helps to– much as I have gone for other issues– I say, have you filled this form? Have you done this? (Interviewer: “You were also helping them.”) Yeah. At the same time, yes, I encourage them.” *Key informant [study coordinator]* Shared values of MJAP and health facility leadership: “…on the whole I think it [START‐ART] is a good public health intervention because it is closer to the test and treat strategy and if we are able to treat many patients I think we can reduce the population viral load and probably have fewer new cases in the community.” (Internist at the national referral and teaching hospital)
Resources	Point of care CD4 cell count machine (PIMA) “The PIMA machine has very much helped us because the type of patients we get here are low income earners. Telling the patient to go back and come back, they see it as a very big problem. Actually, the patient themselves will say, I have come to know whether I can start taking the medicine today. So, the PIMA has very much helped us to ensure that we start our patients on ART in time.” (Nurse at the national referral and teaching hospital) “The clients are satisfied because they don't have to come back like in a week's time, to come and check on their results because of transport issues, they appreciate because they are done there and then. [I: And how do you feel also?] We also feel that we are providing good services.” (Clinician at a small rural HIV clinic) “[The PIMA machine] was received well. As I had told you, sometimes we would lose the results, they would go to Mbarara (a district central laboratory), then you come back after the months that they had given you to come back, review, or the appointment date, you find that your results are not there. So, people received it well…” “Every day if someone is to start ART, he starts. So, it removed that congestion to find that someone is there waiting– and it would bother a lot the counselor and others in education, and so the work is going so well.” (Peer counsellor at the rural, high‐volume HIV clinic) “The PIMA machine… makes our work easy– because those days before the PIMA machine, you would see a patient and you want to have the results, but when it can't come. And, apparently, there was nothing that we could do other than just writing. But now that thing motivates them. You know, when you take a patient to a clinician with all the observations done they will feel so good. He is ready to treat that patient… So now because that machine is there, most patients who go to them have the results, the clinicians are taking the patient's history but at the same time they have the results in front of them. So that motivates them.” (Lead nurse at the national referral and teaching hospital) Training and Coaching “I think these training sessions have helped us because when we received these trainings we got to know what to do and what to emphasize to the patients when starting them on ARVs.” (Nurse at a teaching hospital) “It [training] has increased my knowledge, and to have more motivation to help my colleagues so that they get strong.[…] The health education and patient triage, the way you receive patients and how you pack their drug and give them to them, because all that, they taught us in that training. They taught us nice things.” “… They have helped us, because they stimulate our brains, so that we also know what to do, we get to understand even more.” (Peer counsellor at a small rural clinic) “It [training] helped me analyze how much time these patients spend in the clinic. What is the time they come in and when do they leave? In that time, are we helping them, or they are just seated around or loitering? It helped me assess myself personally, and to ask myself, how many patients am I actually able to see and at what time? And am I actually helping them… I think that is a good thing. (Medical officer at the TB/HIV co‐ management clinic at the national referral and teaching hospital)
Professional role/identity	Strong intrinsic motivation to provide high quality HIV care: “…It is good they thought of [*clinic X*] in this study, because studies usually give an opportunity of learning. The findings can inform or reform decisions, even policies, so we are very grateful… we shall be grateful again if the final feedback is given on the findings – what were the strengths, what were the gaps and maybe missed opportunities, and some recommendations… I mean you just find that you are contributing to someone living an extra day. And also being able to practice what you have studied and maybe you have continued acquiring. Because maybe if you have acquired the knowledge, the skills, but it is not put to use, you don't find that satisfaction.” (Medical officer at a rural high volume HIV clinic) “Seeing my clients appreciating and adhering to the medication and their quality of life improving. That really keeps me moving because you see the product of what you have done, you get to see what your efforts are doing. For example, if they brought someone when they are really sick and after a while they come back jumping and appreciating, there you can say; wow, I am doing a good job… “Each day I see a patient who was in a critical state– I get happy when he is on my table laughing and telling me stories.” (Counsellor at the TB/HIV co‐management clinic at the national referral and teaching hospital) “Usually when we have these patients in the clinic we monitor them for six months and then after we have to transfer them to an HIV care center of their choice. Or if they don't have any we suggest for them. So seeing a patient get better and you are transferring him out of the clinic when he is fine is very rewarding. You feel good that the patient actually came when he was very sick, you have cared for him, and he is fine when he is leaving.” (Clinician in the TB/HIV co management clinic at the national referral and teaching hospital) “…if you go there and educate… in the morning and you see that everyone runs to the lab to go and test, I feel that, that makes me feel good.” (Peer counsellor at a small rural HIV clinic) “The other thing which is rewarding is, we are also creating awareness because when someone undergoes the process of the PIMA machine she is the same person who is going to refer a sister or a friend. They will tell them, go to Mulago and in one day they will work on you, I was also there. You see when these people are together they talk freely; they share and actually empower one another.” (Lead nurse at a teaching hospital) Valorization and respect experienced by HIV‐positive peer educators a powerful motivator: “… If you counsel someone and he meets you and he tells you that “you got me from far that I had gone,” that is the most important part. It is the part that motivates me. You see people respecting you…” “They [clinicians at the facility] appreciate that we are with them.” (Peer counsellor at a small rural HIV clinic) A very personal sense of “mission” to help others, on the part of peer educators: “For me personally I have a love in my heart for the patients, and I told you the reason why I love to help the patients… my husband was sick and bedridden for two years and yet he was the one who was working by then, I was not working. But for these two years he was bedridden, it was really terrible and I had given up on him and I started praying to God to take him. But miraculously my husband came up [to the clinic] and he got well and he is fine today. So because of what I saw that happened to my husband, I decided that I will never ignore a patient no matter how worse they come in at the clinic here.” (HIV peer counsellor at a teaching hospital)

MJAP, Makerere University Joint AIDS Program; TDF, Theoretical Domains Framework; START‐ART, Streamlined antiretroviral therapy.

### 2.6 Ethical issues pertaining to human subjects

Individual informed consent was obtained from all individual patients and providers who participated in the qualitative study. The study received ethical approval from the ethical research committees of the School of Medicine at Makerere University College of Sciences, the Uganda National Council for Science and Technology and the University of California San Francisco.

## 3 Results

The findings presented below indicate how new practices at facilities were rapidly disseminated through a network beginning from the Ugandan HIV programme, to facility health workers, and into peer counsellors. The dissemination of these new practices was enabled by the *environmental context* for START‐ART intervention, MJAP, which provided a strong foundation of shared purpose and accountability that enabled uptake of the intervention. MJAP's features may have facilitated *social influences* on facility managers and the front line providers they oversee via lines of communication and oversight. The comments of providers interviewed demonstrated a sense of shared identity and purpose within this network structure, and revealed both a sense that the providers felt that their accomplishments were recognized and that their actions were monitored. As such, they reported a sense of accountability to MJAP and Ministry of Health leadership. Some expressed hope this could facilitate a national expansion of the intervention beyond the study facilities. As an internist at the national referral and teaching hospital told us, “… If you are doing it in MJAP‐supported clinics I think it's good. My thinking is that it should expand. I am sure your results are going to be good and my thinking is that it should expand to include all the other centers where people go, even people who are not attending public facilities.”

The fact that START‐ART study team members were largely not hired de novo, but rather were existing MJAP technical team members already engaged in providing technical support and assistance to clinics, may have also facilitated uptake of the intervention. This may have inculcated a sense of solidarity and support for the front line providers. A study coordinator, in a key informant interview conducted by the U.S.‐based project leader, reported that he often “pitched in” with clinical duties to share the clinical care burden with providers with heavy workloads, as a part of scheduled study monitoring visits. This engendered a sense of trust that facilitated intervention uptake; moreover, monitoring visits that could have been perceived as threatening or evaluative were instead welcomed by the front line providers.

The *resources* provided by MJAP for this study, posited by TDF to be intertwined with other aspects of the environmental context, included the coaching and monitoring provided by study team members. This alone facilitated providers’ motivation. As a medical officer at the national referral and teaching hospital commented, “…if it is done in a way that after three months you come and check on us officially, and ask us, how are you doing? It keeps us going.” Other resources included specific skills training; providers were proud of attainment of new knowledge and skills and gratified by providing higher quality care, as illustrated by the comments shown in Table 1. Secondly, of all resources provided by the study, providers viewed the provision of PIMA machines as the most instrumental to the success of the intervention. As illustrated in the comments in Table 1, the use of PIMA relieved providers from frustrations of lost or delayed CD4 cell count results and led to higher patient satisfaction, via providing immediate results and reducing the need for pre‐ART visits to the clinic. As a medical officer commented, “the PIMA machine itself […][was] also motivating because it offers timely intervention which the patient needs.” This new resource gave a morale boost to providers, which may have helped them overcome any reticence to accept changes in practice:

“You see changes are also not that easily welcomed. […] Because, everyone was wondering, how is it going to benefit us? But now we have got the benefits that patients are now starting there and then, and we have reduced on the number of patients waiting to be started on ART.” *Counsellor at the TB/HIV clinic at the national referral and teaching hospital*.

On a practical level, the PIMA helped to overturn long‐standing norms that reinforced delays in ART initiation:

“The immediate ART initiation […] is something that we were already practicing […] But there would be those who have their reasons and they are not ready to begin– you wouldn't really force them. Or, there was this issue of somebody waiting for the CD4 if they don't meet the […] eligibility criteria. So we would give them a return date of a month or two weeks depending on how physically they are, because you have to receive the CD4. But given that there is PIMA, you can have the CD4 immediately […] it has increased on those started on ART immediately…” *Medical officer at a rural high ‐volume HIV clinic*.

Finally, findings of the study support the notion, as posited by the TDF, that providers’ *social or professional role and identity* played a role in facilitating the uptake of accelerated ART initiation. The front line clinicians offering care to HIV patients appeared to demonstrate a strong sense of mission to provide high quality care. For these providers, patient satisfaction was a motivator, as was seeing patients get better, as illustrated in the quotes shown in Table 1. A medical officer at the national referral and teaching hospital offered this narrative that exemplifies this intrinsic motivation:

“Seeing patients improve I think is the most paying thing you can ever get. Patient brought in bedridden but stands on his feet and walks back home, that is enough. There is that inner feeling of satisfaction. And you may think you are helping only one person, the patient, but when you are helping so many people who are around that person. The children, the relatives, and of course some of our patients are the breadwinner in the family.”

An unforeseen finding was that peer counsellors (patients successfully engaged in HIV care, who volunteer at health facilities by providing pre‐test counselling and information about HIV care at the facilities) were reached by the START‐ART intervention, adopted it readily, and also shared a strong sense of *professional/social identity* related to their role in the health system. Comments from IDIs across data sources suggest that peer counsellors served as important role models for other patients and were advocates of early ART initiation. For these individuals who often experience forms of HIV‐related stigma 16, 17, the role of peer counsellor may provide a powerful means of obtaining a renewed respect and valorization in local communities.

“Although MJAP provided a modest monthly stipend to the peer counselors, the bigger driving factor is, there is prestige in being like a health worker… now people in the village they are seeing you, now you are the one who is at the forefront of some activity in the health centre. So there is that prestige.” *Key informant [study coordinator]*.

Peer counsellors frequently recounted that their sense of mission was driven by their personal experiences of having benefited from HIV care and treatment. The respect and gratitude that patients expressed also powerfully motivated peer counsellors. Unlike the conversations between clinicians and patients, peer educators often recounted conversations they had with their fellow community members outside of the clinic setting, underscoring the important status they felt they had gained in the community through their helping role in clinics.

Peer counsellors may have played an instrumental role in the START‐ART intervention, despite the fact that they were not in any way specifically targeted for inclusion in the intervention design or delivery. A peer counsellor at a small rural clinic commented, “they [clinicians at the facility] appreciate that we are with them.” In turn, clinicians spoke positively of the role of the peer counsellors in the care system. As a clinician in the national referral and teaching hospital commented, “these [peer counselors], I believe spend much more time with the patients than the doctors.” Finally, a component of the STARTs intervention that was intended to reinforce provider uptake of the novel practices was the provision of bi‐annual clinic feedback cards, which gave providers an overview of their progress in timely initiation of ART for patients. This intervention component did not, in qualitative interviews, figure prominently as a major influence or source of motivation for the providers.

## 4 Discussion

Qualitative findings from the START‐ART study highlighted the importance of institutional context for enabling the diffusion of new evidence‐based practices: strong existing relationships at the institutional and interpersonal level enabled a rapid diffusion of innovative practices in this study. MJAP had a strong history of partnership with facilities, and this relationship in turn enabled coordinators and opinion leaders from the implementing organization to influence front line health workers through information, training and coaching. An already existing professional link between MJAP technical staff and the HIV providers at the facilities provided a good environment for transfer and acceptance of the evidence on the dangers of delayed ART initiation for eligible individuals. A second enabling factor was the strong intrinsic motivation among providers to provide high quality care, and therefore to adopt new practices perceived as beneficial to patients. This strong professional role/identity of providers facilitated the adoption of new knowledge, but its ready acceptance was enabled by an environmental context characterized by communication and accountability from the HIV programme's technical staff to front line providers.

In the global response to HIV, non‐governmental organizations (NGOs) led by nationals have played an important supportive role in the scaling up HIV services. NGOs have institutional advantages through unique characteristics such as: smaller size, more flexible administrative systems, and closer links to the front line health workers and communities that enable them to be innovative and nimble 18. NGOs providing technical support for HIV services have been widely used but little systematic appraisal has been documented. Evidence from a large ecological analysis suggested the strength of NGOs was associated with reduced HIV prevalence in more democratic settings 19. The present study suggests that NGO such as MJAP may be a particularly important “actor” 20 in innovations in the global response to HIV.

PIMA machines significantly reduced the turnaround time for the CD4 cell count results and enabled same day determination of ART eligibility. Providers felt pride and accomplishment with attainment of new knowledge and skills, and PIMA machines allowed them to give immediate care rather than sending patients away. While these POC CD4 cell count machines are not recommended at high volume facilities in Uganda, their use facilitated rapid uptake of ART irrespective of facility size. Although the POC CD4 test has not been found to improve ART uptake as a sole intervention 21, in the context of other predisposing and reinforcing factors, it contributed to a twofold increase in the number of patients who initiated ART 14 days after ART eligibility as compared to the standard of care. These findings favour the use of multifaceted approaches informed by an implementation framework, rather than single‐intervention approaches.

Although not directly targeted during the knowledge transfer phase of the study, peer counsellors who attended the training sessions with the clinical staff played a critical role as enablers of rapid ART initiation among eligible patients, and this study confirms their critical importance in the HIV response 22, 23. Peers transmitted new beliefs and attitudes about rapid HIV treatment initiation to communities and patients, and thereby facilitated an increased demand for this new practice. Peer counsellors reported that the patients could easily identify with the personal experiences that they shared, which quickly built patients’ belief and trust in the medicines that were being recommended by the clinicians, suggesting that they played a key role in influencing patients to start on ART. In turn, the peer counsellors felt empowered by attaining knowledge of the benefits of early ART initiation and, overall, respected by colleagues. More importantly, they were motivated not only by the small remuneration for their work but by altruistic desire to help others, and an elevated stature in communities. Clinicians felt the peer counsellors prepared patients well for their often much briefer interactions with patients. The effect was most felt at lower level facilities, which are staffed with fewer health providers. Peer counsellors played a more central role in patient care in such settings.

There were limitations to this study. It was conducted in a setting with an established collaboration between the organization that provided technical assistance (MJAP) and health care providers at the health facilities; and, although this is a common characteristic of HIV service delivery in resource limited settings, it may not be uniformly similar and therefore our findings may not be generalizable to all settings. Uganda differs socio‐culturally from neighbouring countries in the region, and therefore, the effect of peers who are based at a facility on community members may not be entirely transportable. Finally, these findings are derived from interviews with the total but small number of providers who are responsible for direct care delivery at the five clinics participating in Wave 3 of the trial. The interview narratives provided rich, in‐depth perspectives from these providers on the intervention and on other aspects of care provision, but because of the small number of clinic settings and providers, we are constrained in our ability to pursue comparisons across sites and cadre. To keep the manuscript to publishable length, we have not included secondary findings, deviant case analyses or smaller exceptions and contradictions in the data, and have limited our discussion of pre‐intervention findings. We instead have focused this paper on the broader emergent themes for which there were ample supportive data.

In conclusion, as intended, START‐ART reduced barriers to the provision of high quality care. As suggested by the TDF, findings point to multiple dimensions of influence of uptake of new evidence‐based practice, including contextual factors in the health systems environment, a strong professional identity on the part of not only clinicians but also of a largely volunteer cadre, and resources offered by START‐ART including both tangible technological tools (PIMA) and the training and coaching that facilitated uptake of this new technology. Uptake of the START‐ART intervention also was mediated by unanticipated factors: persuasive peer counsellors provided a set of evidence for patients as models for successful ART uptake, before patients interacted with the clinicians. These influences facilitated patients’ readiness to initiate ART quickly, often on same day eligibility was determined. The findings of this study offer positive insights for the uptake of test and treat approaches for HIV in similar settings. We conclude with two recommendations. First, for researchers seeking to understand implementation processes, qualitative methods are essential for understanding unknown unknowns, which are expected in implementation research. Second, for implementers involved in the global response to HIV, we suggest that effective health systems innovations to optimize health outcomes often occur at the intersection of health systems and communities. The incorporation of people living with HIV as peer counsellors in the health care workforce represents a generalizable strategy to naturally bridge and connects systems and communities.

## Competing interests

The authors declare that they have no competing interests.

## Authors’ contributions

FCS, CC, GA, JN, MRK DH, EC and EG conceived and designed the original study; FCS, CC, JW, LK, RK and EG analysed the data; FS, CC, JW, LK and EG drafted the manuscript; EG, DH, GA, JN, JK, WC and MRK critically reviewed the manuscript. All authors read and approved the final manuscript.

## Funding

Research reported in this publication was supported by the NIH/NIAID under awards U01AI099959, P30 AI027763, K24 AI134413 and Fogarty International Center of the National Institutes of Health under award number D43 TW010037. The content is solely the responsibility of the authors and does not necessarily represent the official views of the National Institutes of Health.
